# Efficient Fluoride Wastewater Treatment Using Eco-Friendly Synthesized AlOOH

**DOI:** 10.3390/nano13212838

**Published:** 2023-10-26

**Authors:** Wan-Tae Kim, Joo-Won Lee, Hong-Eun An, So-Hye Cho, Sohee Jeong

**Affiliations:** Materials Architecturing Research Center, Korea Institute of Science and Technology, Seoul 02792, Republic of Korea; wantaekim@kist.re.kr (W.-T.K.); lee2080@kist.re.kr (J.-W.L.); ahe9930@kist.re.kr (H.-E.A.); sohyec@kist.re.kr (S.-H.C.)

**Keywords:** AlOOH, wastewater, fluoride ion removal

## Abstract

Fluoride ion is essential for health in small amounts, but excessive intake can be toxic. Meeting safety regulations for managing fluoride ion emissions from industrial facilities with both cost-effective and eco-friendly approaches is challenging. This study presents a solution through a chemical-free process, producing a boehmite (AlOOH) adsorbent on aluminum sheets. Utilizing cost-effective Al foil and DI water, rather than typical precursors, yields a substantial cost advantage. The optimized AlOOH adsorbent demonstrated a high fluoride ion removal rate of 91.0% in simulated wastewater with fluoride ion concentrations below 20 ppm and displayed a similar performance in industrial wastewater. Furthermore, the AlOOH adsorbent exhibited excellent reusability through a simple regeneration process and maintained stable performance across a wide pH range of 4 to 11, demonstrating its capability to adsorb fluoride ions under diverse conditions. The efficiency of the AlOOH adsorbent was validated by a high fluoride ion removal efficiency of 90.9% in a semi-batch mode flow cell, highlighting its potential applicability in engineered water treatment systems. Overall, the AlOOH adsorbent developed in this study offers a cost-effective, eco-friendly, and sustainable solution for effectively removing fluoride ion from surface waters and industrial wastewaters.

## 1. Introduction

Fluoride is a common element found in various minerals and widely used in various industries [1,2,3]. It has numerous benefits for human beings, such as reducing enamel erosion and preventing osteoporosis at low concentrations. However, prolonged exposure to high levels of fluoride ions can lead to dental and skeletal fluorosis and other related diseases [4,5,6]. To ensure safe exposure to fluoride ions, the World Health Organization (WHO) has recommended a limit of 1.5 ppm or less of fluoride ions in drinking water [4,7]. Additionally, some jurisdictions recommend a limit of 2.0 ppm or less of fluoride ions in wastewater [8]. Wastewater contaminated with fluoride ions is increasingly being discharged from various industrial processes such as semiconductor manufacturing and fertilizer industries [9,10]. In order to comply with safety regulations and restrict imprudent fluoride ion emissions from industrial facilities, fluoride ions have been captured and precipitated in the form of CaF_2_ using Ca(OH)_2_ as a coagulant aid. However, even after fluoride ion removal via precipitation, water can still contain fluoride ion levels of about 20 ppm that exceed the effluent quality standard [11], which requires additional post treatments.

To meet the requirements for drinking water and wastewater, various methods including chemical precipitation [12], ion exchange [13], electrochemical processes [14], and adsorption techniques [15,16,17] have been developed to remove excess fluoride ions [7]. Adsorption is one of the most attractive options due to its low cost and simplicity of operation. Various materials have been examined as adsorbents for fluoride ion removal, such as activated carbon, metal oxides, and Al-based materials. Although activated carbon and metal oxides are cost-effective options for large-scale treatment systems, their effectiveness in fluoride ion removal is limited [18]. In contrast, Al-based fluoride ion adsorbents have been extensively studied due to their strong affinity to fluoride ion and their cost-effectiveness in large-scale operations. This preference for Al-based fluoride ion adsorbents can be explained by Pearson’s Hard Soft Acids Bases (HSAB) theory, which states that the Al ion (a hard Lewis acid) has a strong affinity for fluoride ions, as the fluoride ion is the hardest Lewis base with the highest electronegativity and a small ionic radius [19,20,21].

Boehmite (AlOOH) is a prominent representative Al-based mineral that is not only cost-effective and abundantly available but also easy to synthesize. Notably, AlOOH has a relatively strong adsorption ability for fluoride ions compared to other Al-based materials, such as gibbsite (Al(OH)_3_) and activated Al_2_O_3_ [3,4,22]. The hydroxyl ions (OH^−^) on the surface play a crucial role in achieving high fluoride ion removal efficiency by facilitating the exchange of hydroxyl ions for fluoride ions. Consequently, boehmite and gibbsite, which have a relatively large amount of Al-OH bonds, exhibit a higher fluoride ion removal capacity compared to activated Al_2_O_3_. Despite the higher concentration of Al-OH bonds in Al(OH)_3_, AlOOH has a better fluoride ion removal performance due to its higher stability in response to pH changes in water [23,24]. Ensuring high performance across a wide range of pH levels is crucial, as these levels can vary depending on the environment of various wastewaters.

The type of sorbents plays a pivotal role in designing a fluoride ion removal process that is both cost-effective and high-performing. Sorbents can be categorized into powder or granular forms, with powder adsorbents being the most widely employed for fluoride ion removal from wastewater. However, this method presents economic drawbacks due to its limited recyclability and substantial space requirements. Additionally, after fluoride ion removal, the powder adsorbents must undergo filtration prior to discharge, resulting in the generation of significant amounts of sludge. The proper disposal of the sludge can be difficult and expensive [5,25]. Moreover, the improper collection of the sludge during water treatments can lead to the release of the powder adsorbents into the environment, posing a significant threat to human health [7,26,27]. To address these concerns associated with the release of adsorbent powders, researchers have explored the utilization of various substrates, such as polyacrylonitrile nanofibers [28], polyaniline nanofibers [29,30], and cellulose nanofibers [31], to create composite adsorbents—where powder adsorbents are fixed on a substrate. However, the fabrication of these composite adsorbents often involves metal precursors, organic solvents, and other chemicals, such as anionic metal salts, acetone, hydrochloric acid, and ammonia solution, which can have adverse environmental effects. Therefore, it is crucial to develop a cost-effective and recyclable adsorbent that can be produced through environmentally friendly processes.

**Scheme 1:**
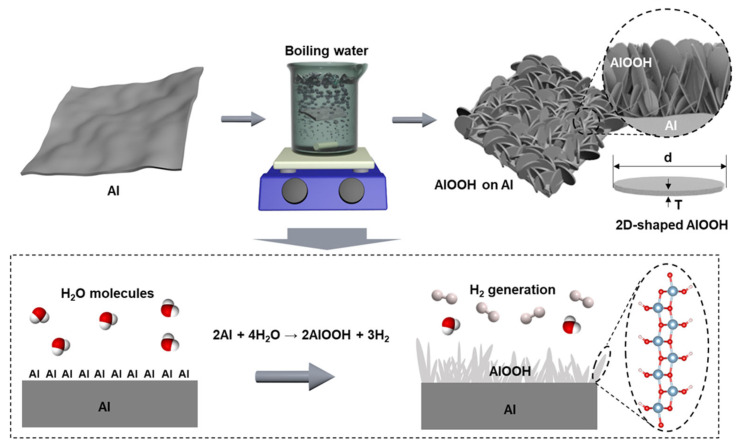
A cost-effective, eco-friendly, and simple method for AlOOH fabrication on the surface of Al foil using boiling DI water. The 2D-shaped AlOOH nanostructures within the AlOOH film show a high ratio of lateral size (d) to thickness (T).

Herein, an efficient adsorption of fluoride ions is presented using a 2D-shaped AlOOH-based adsorbent grown on aluminum (Al) foil. Our fabrication method is simple, cost-effective, and environmentally friendly, involving only the reaction solely between the Al foil and boiled DI water (Scheme 1). By controlling the immersion time of Al in boiling DI water, the fabrication process is optimized to achieve an adsorbent with a high fluoride ion removal rate. The fluoride ion adsorption properties of the optimized AlOOH are evaluated using the pseudo-first-order and Langmuir isotherm model. The effectiveness of the adsorbent in removing fluoride ions at low concentrations ranging from 5 to 20 ppm and a wide range of pH levels (4, 5, 7, 9, and 11) is demonstrated. To evaluate the reusability of the optimized AlOOH, cycling experiments are carried out that illustrate its sustained fluoride ion removal capacity through a regeneration process. Additionally, two distinct experiments are performed to emulate practical wastewater treatment systems. First, the fluoride ion removal rates of AlOOH are tested using actual industrial wastewater. Second, the fluoride ion removal performance is verified by operating a continuous flow cell in a semi-batch mode with the AlOOH fabricated Al mesh, achieving a high fluoride ion removal efficiency of 90.9%.

## 2. Materials and Methods

Commercial Al foil (household Al foil) is used in order to fabricate AlOOH, as it is immersed in boiling DI water for various durations ranging from 10 s to 4.0 h. The surface morphology, chemical compositions, and crystal structure were investigated via a field emission scanning electron microscope (FE-SEM, Inspect F, FEI Company Co., Ltd., Hillsboro, OR, USA), energy-dispersive spectroscopy (EDS), a transmission electron microscope (TEM, Titan, FEI Company Co., Ltd.), and Fourier transform infrared spectroscopy (FT-IR, Nicolet iS10, Thermo Fisher Scientific Co., Ltd., Boston, MA, USA). The cross-sectional view of AlOOH fabricated on Al foil was observed via FE-SEM and TEM after the preparation of the sample using ion milling and a focused ion beam (FIB). The chemical composition of AlOOH adsorbent was measured via X-ray photoelectron spectroscopy (XPS, Nexsa, Thermo Fisher Scientific Co., Ltd.) with Al Kα X-ray radiation as the X-ray source for excitation and FT-IR with diamond attenuated total reflection (ATR).

The concentration of fluoride ions in simulated wastewater was adjusted with hydrofluoric acid (HF, 40%, Alfa Aesar Co., Ltd., Boston, MA, USA). Fluoride ion removal rates of AlOOH were confirmed using sodium 2-(parasulfophenylazo)-1,8-dihydroxy-3,6-naphthalene disulfonate (SPADNS) and zirconium oxychloride, along with a colorimeter (HI-739, HANNA instruments Co., Ltd., Seoul, Republic of Korea). Its maximum fluoride ion adsorption capacity was evaluated using a pseudo-first order and Langmuir isotherm model.

The pH of each solution was adjusted with small drops of aqueous solutions containing nitric acid and sodium hydroxide. For the reusability test the fluoride ion adsorbed sample was repeatedly immersed in boiling DI water (5 times). Furthermore, the fluoride ion removal rates in diluted semiconductor wastewater were assessed, which was initially at a concentration of 5000 ppm and then diluted with DI water to a concentration of 20 ppm. For the evaluation, AlOOH was fabricated on the surface of a porous Al mesh (2.0 mm × 2.0 mm mesh, 0.24 mm diameter) and the fluoride ion removal rate of the AlOOH fabricated sample was then evaluated within the flow cell using 1.00 g of the sample in 25 mL of diluted semiconductor wastewater. The flow cell consists of a sample container (with an inner diameter of 4 cm), a peristaltic pump, simulated wastewater, and tubing. Two sample containers were connected to accommodate the loading of Al mesh samples.

## 3. Results and Discussions

### 3.1. Synthesis and Characterization of AlOOH Adsorbents on Al Substrate

The sheet-type AlOOH adsorbent was synthesized via eco-friendly and cost-effective methods using Al foil and DI water. The significant cost advantage of this method is emphasized by the fact that aluminum foil is up to 90 times less expensive than a typical aluminum precursor and it eliminates the need for any organic solvents (see Table S1). The foil was immersed in boiling DI water, resulting in the formation of AlOOH on the surface, which was used for the fluoride ion removal test without any rinsing and activation of adsorption sites. AlOOH is generated when Al on the surface of the foil reacts with water molecules during boiling, resulting in the deposition of AlOOH on the Al surface and the concurrent generation of hydrogen gases as a by-product (Scheme 1) [32,33,34]. The resulting samples were designated as AlOOH-10s, AlOOH-30s, AlOOH-1m, AlOOH-5m, AlOOH-0.5, AlOOH-1, AlOOH-2, and AlOOH-4 corresponding to the immersion time of 10 s, 30 s, 1 min, 5 min, 0.5 h, 1.0 h, 2.0 h, and 4.0 h, respectively. The color of the foil transitioned from shiny silver to matte silver mixed with white, indicating the growth of AlOOH on the Al foil (see Figure S1a,c,e,g) [35].

**Figure 1:**
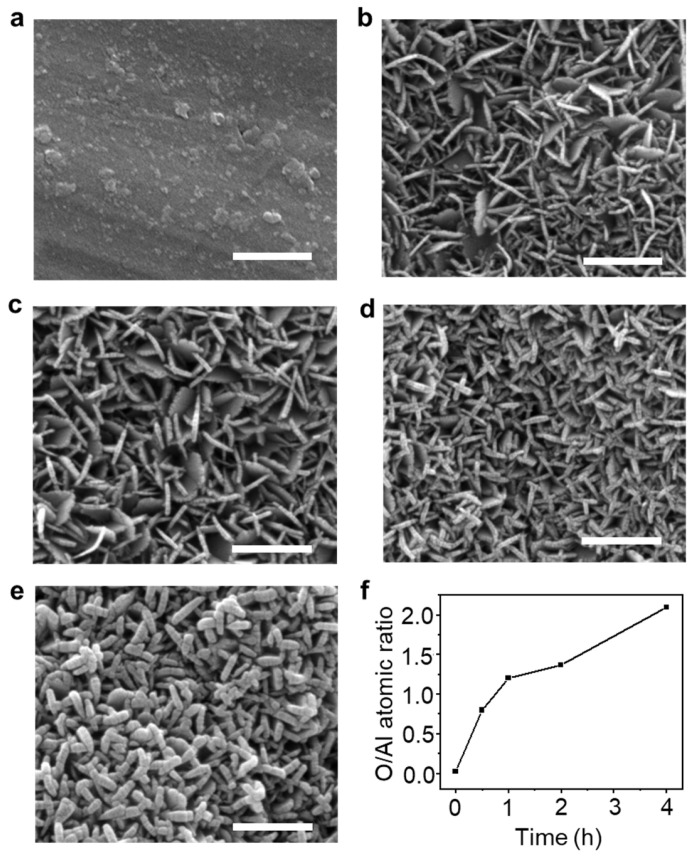
FE-SEM images of Al foil (**a**) before and (**b**–**e**) after immersion in boiling DI water with different immersion times ((**b**): 0.5 h, (**c**): 1.0 h, (**d**): 2.0 h, and (**e**): 4.0 h, refer to AlOOH-0.5, AlOOH-1, AlOOH-2, and AlOOH-4, respectively; white scale bar is 500 nm), and (**f**) Al and O atomic ratio of (**a**–**e**), which was analyzed via EDS analysis.

The FE-SEM images show the morphology of as-synthesized AlOOH on the Al surface. Two-dimensional (2D)-shaped Al-O compounds were grown more densely on the Al surface with the increase in immersion time from 10 s to 1 min, as shown in Figure S2a–c [35,36]. Moreover, the thickness of AlOOH increases with longer immersion times in boiling DI water, measured to be about 21 nm, 27 nm, 30 nm, and 59 nm for AlOOH-0.5, AlOOH-1, AlOOH-2, and AlOOH-4 (the AlOOH series), respectively (as shown in Figure S2d and Figure 1b–e). This increase was even more pronounced in AlOOH-4, leading to a substantially thicker AlOOH which entirely covered the surface (see Figure 1e). In order to accurately measure the height of the AlOOH film on the Al foil, a side view of samples prepared via ion milling was observed via FE-SEM. As a result, the height of the AlOOH film increased with longer immersion times, measured to be about 483 nm, 501 nm, 504 nm, and 602 nm (Figure S1b,d,f,h), respectively. Overall, AlOOH shows superior growth in a direction parallel to the lateral direction, resulting in a high aspect ratio of lateral size to thickness.

The elemental composition in both the top view and the cross-sectional view was determined via EDS analysis (Figure 1f and Figure S3, Tables S1 and S2). The O/Al ratios for the AlOOH series were determined to be 1.90, 1.89, 1.96, and 1.97, respectively. These values were calculated based on the analysis from a cross-sectional view, where solely AlOOH was detected, unlike the top view. These ratios are consistent with the theoretical O/Al ratio for AlOOH, confirming the formation of AlOOH on the surface (Table S2). The trend in O/Al ratios in the AlOOH series is also observed as show via the top view, where both AlOOH and Al foil are detected. The O/Al ratio exhibits a rapid increase within the first 5 min of the reaction, followed via a slower rate of increase with a gentler slope by top-view EDS analysis. This suggests that Al-O clusters rapidly form within a few minutes, leading to a sudden increase in oxygen concentration [37]. Subsequently, as these Al-O clusters transform into AlOOH, there seems to be a decrease in the increment of the O/Al ratio.

**Figure 2:**
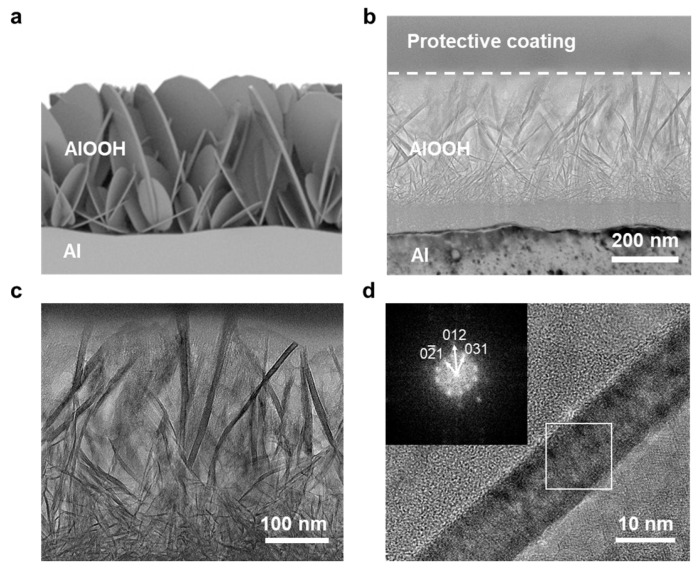
(**a**) Illustration of AlOOH-2 on Al foil and TEM images of AlOOH-2: (**b**–**d**) cross-sectional TEM image of AlOOH on the Al surface (inset showing the FFT pattern of the white square box region).

TEM analysis was performed to observe the more detailed morphology and crystal structure of AlOOH on the surface of Al. The observation revealed the presence of 2D-shaped AlOOH structures with a lateral size of less than 600 nm on the Al surface (Figure 2b,c). A schematic illustration of AlOOH on the Al surface is provided in Figure 2a, corroborating the finding via the cross-sectional view obtained from FE-SEM. In Figure 2d, the fast Fourier transform (FFT) pattern of AlOOH-2 showed the presence of (0 −2 1), (0 1 2), and (0 3 1) planes of AlOOH [38], indicating that AlOOH was successfully fabricated on the Al surface.

**Figure 3:**
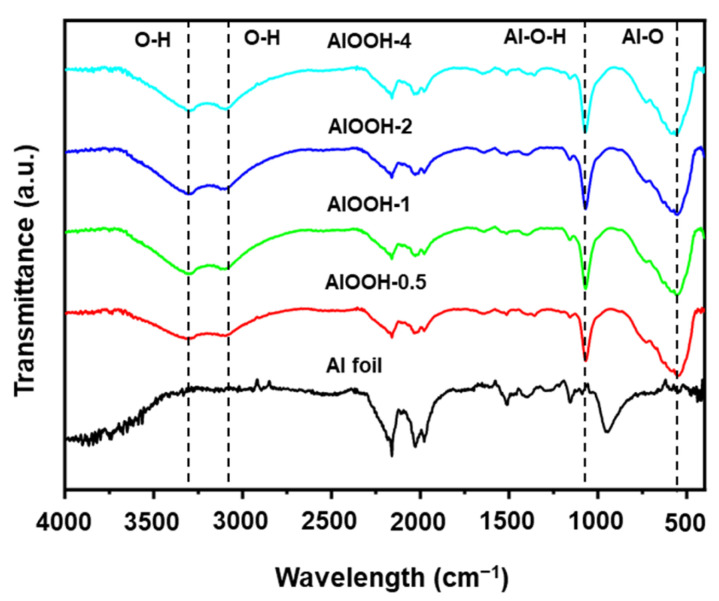
FT-IR spectra of Al foil, AlOOH-0.5, AlOOH-1, AlOOH-2, and AlOOH-4.

FT-IR data also provide evidence for the formation of AlOOH on the surface of Al (Figure 3). Compared to Al foil, the FT-IR spectra of the AlOOH series exhibited two distinct bands at 3301 and 3096 cm^−1^ which are attributed to O-H bonds from the physically adsorbed water [3] in hydrophilic AlOOH. In addition, the bands corresponding to Al-O-H and Al–O bonds of AlOOH are observed at 1063 cm^−1^ and 594 cm^−1^, respectively [37,39,40].

### 3.2. Fluoride Ion Removal Capacity of AlOOH Adsorbents on Al Substrate

**Figure 4:**
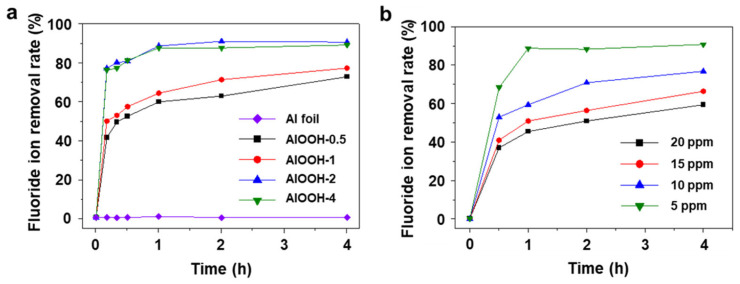
Fluoride ion removal rate with (**a**) different immersion times in boiling water (Al foil, AlOOH-0.5, AlOOH-1, AlOOH-2, and AlOOH-4, initial fluoride ion concentration: 20 ppm, sample loading: 1.00 g, and adsorption time: 4.0 h) and (**b**) different initial fluoride ion concentrations ranging from 5 to 20 ppm of AlOOH-2 (sample loading: 0.25 g, and adsorption time: 4.0 h).

With successfully fabricated AlOOH adsorbents, the fluoride ion adsorption capacity is initially evaluated using the simulated wastewater with 25 mL of 20 ppm HF solution. The choice of 20 ppm of fluoride ion as the initial concentration is made because conventional adsorbents often struggle to reduce fluoride ion levels below this point. For example, at concentrations lower than 20 ppm, the efficiency of the method utilizing Ca(OH)_2_ as a coagulant aid for adsorbing fluoride ions tends to decrease because of the dissolution of the adsorbed form of F ions (CaF_2_) back into the solution [11,41]. To find the most effective adsorbent among AlOOH-0.5, AlOOH-1, AlOOH-2, and AlOOH-4, the fluoride removal rate was measured using a colorimeter (Figure 4a). The rate of AlOOH-2 and AlOOH-4 reached steady states within 1.0 h, with removal rates of approximately 91.0% and 89.5%, respectively. The AlOOH-2 sample shows a decrease in fluoride concentration from 20 ppm to 1.8 ppm, showing compliance with the regulatory standards (2.0 ppm) [8]. Unlike AlOOH-2, for AlOOH-0.5 and AlOOH-1, the fluoride ion removal rate did not reach a steady state until 4.0 h, with fluoride ion removal rates of 73.0% and 77.5%, respectively. Among the samples, AlOOH-2 exhibited the highest adsorption performance. Therefore, AlOOH-2 was chosen as an efficient adsorbent for the fluoride ion adsorption tests at concentrations below 20 ppm (Figure 4b). To better discern efficiency differences under these low concentration conditions, the weight of the adsorbent was reduced in the test. After 4.0 h, the fluoride ion removal rates were 91.0%, 77.0%, 66.5%, and 59.5% at 5 ppm, 10 ppm, 15 ppm, and 20 ppm, respectively. These results indicate that AlOOH-2 is an effective adsorbent even at low fluoride ion concentrations (below 20 ppm).

**Figure 5:**
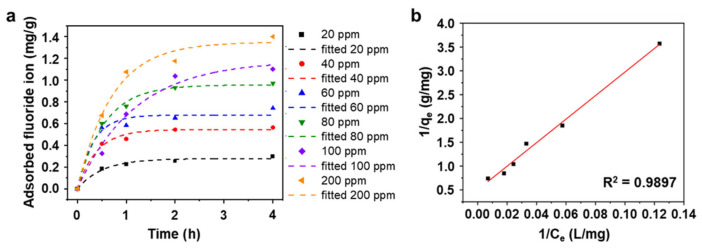
(**a**) Fluoride ion adsorption kinetics of AlOOH-2 with different initial fluoride ion concentrations (dashed line shows the result of fitting a pseudo-first-order model with each initial concentration of fluoride ions) and (**b**) isotherm of fluoride ion adsorption of (**a**) with linear form Langmuir isotherm model. (Initial fluoride ion concentration: 20 to 200 ppm, sample loading: 0.25 g, and adsorption time: 4.0 h.).

To further understand the maximum capacity of AlOOH-2 for fluoride ion adsorption, kinetic studies are conducted as shown in Figure 5a and Table S3. For the fluoride ion removal adsorption tests, 0.25 g of the sample is soaked in 25 mL of HF solution. Measurement of the rate of fluoride ion adsorption was carried out using varying initial concentrations (ranging from 20 to 200 ppm) in a simulated wastewater environment. At an initial fluoride ion concentration of 20 ppm, the fluoride ion adsorption rate reached a plateau of 0.30 mg/g. For higher initial concentrations (40 ppm, 60 ppm, 80 ppm, and 100 ppm), the adsorption rates reached steady states after 2.0 h, measuring 0.56 mg/g, 0.74 mg/g, 0.97 mg/g, and 1.10 mg/g, respectively. In the case of 200 ppm of simulated wastewater, the adsorption rate took a longer time to reach a steady state, stabilizing after 4.0 h at 1.40 mg/g. Consequently, the total fluoride ion removal rates increased as the initial fluoride ion concentration decreased (Figure 5a and Figure S4), indicating once again that our absorbent is more effective at lower fluoride ion concentrations in wastewater. Figure 5b shows the Langmuir adsorption isotherm fitting of AlOOH on Al foil with different initial fluoride ion concentrations and adsorption times, illustrating the process of fluoride adsorption. Utilizing the pseudo-first order model and the Langmuir isotherm model, the maximum fluoride ion adsorption capacity (q_max_) of AlOOH-2 is calculated to be 1.98 mg/g based on the total sample mass (Al and AlOOH), with additional details provided in text of the Supplementary Materials and Tables S3 and S4 [22,42]. It is important to note that AlOOH, which solely undertakes the role of an adsorbent, only exists on the surface of the Al foil which is an inactive or dead adsorbent. AlOOH comprises 6.15 vol% (the volumetric ratio, calculated from the height of the AlOOH film, as shown in Figure S1) and 7.58 wt% (calculated from the volumetric ratio and molar mass of Al and AlOOH, assuming that the AlOOH layer consists of dense layers) of the total sample (Al and AlOOH). The fluoride ion adsorption isotherms of AlOOH-2 are normalized using the weight percent of AlOOH in the total sample (Figure S5). Consequently, the maximum adsorption capacity of fluoride ions can be normalized to 26.12 mg/g based on the weight of AlOOH in the sample. This capacity is comparable to values reported in previous studies, even though AlOOH-2 shows lower total weight-based capacities compared to other Al-based materials (Figure S6).

### 3.3. Fluoride Adsorption Mechanism of AlOOH Adsorbents on an Al Substrate

**Figure 6:**
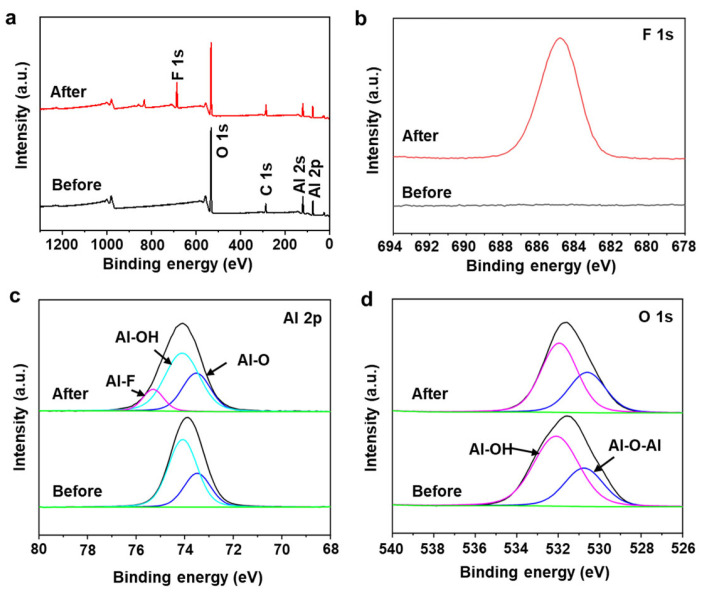
(**a**) XPS survey spectra and XPS spectra of (**b**) F 1s, (**c**) Al 2p and (**d**) O 1s of AlOOH-2, before and after fluoride ion removal (green: background, black: experimental data).

**Figure 7:**
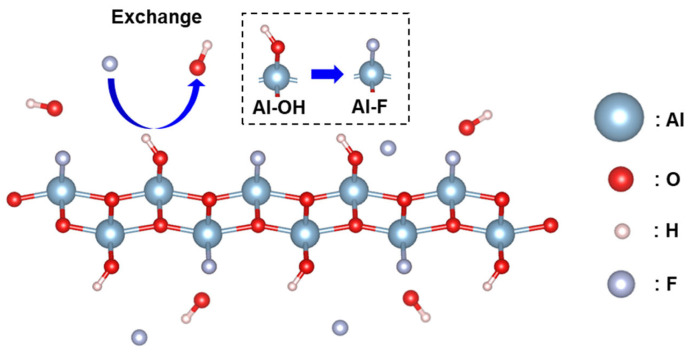
Schematic illustration of fluoride ion adsorption on AlOOH-2.

To understand the mechanism of fluoride ion adsorption, the reaction process is investigated by analyzing the XPS and FT-IR spectra of the AlOOH-2 before and after fluoride ion adsorption. To maximize the fluoride ion adsorption, AlOOH-2 (mass = 1.00 g) was immersed in a 25 mL 200 ppm HF solution for 4.0 h. After fluoride ion adsorption, new peaks corresponding to adsorbed fluoride species were observed at 684.9 eV in the F 1s spectrum and 75.3 eV, in the Al 2p spectrum, confirming the presence of fluoride ions on the surface of AlOOH-2 (Figure 6a,b) [3,43,44]. Additionally, the content of Al-OH, located at 74.1 eV decreased from 68.36 to 56.87% after fluoride ion adsorption (Table 1). In the O 1s spectra, the peak at 532.0 eV, which corresponds to Al-OH, also showed a decreasing trend from 68.03 to 63.04% (Table 1) [3]. The decrease in Al-OH bonding in AlOOH-2 and the formation of Al-F bonding indicated that the hydroxyl ions on the surface serve as active sites for fluoride ion adsorption and facilitate the exchange between hydroxyl ions and fluoride ions (Equation (1) and Figure 7) [3,45,46]. Also, the involvement of hydroxyl ions in the fluoride ion adsorption process was confirmed via FT-IR analysis of the AlOOH-2 before and after fluoride ion removal (details are shown in the text of the Supplementary Materials and Figure S6).

F^−^ + Al-OH → Al-F + OH^−^ (1)

### 3.4. pH Variance, Reusability, and Industrial Wastewater Evaluation

**Figure 8:**
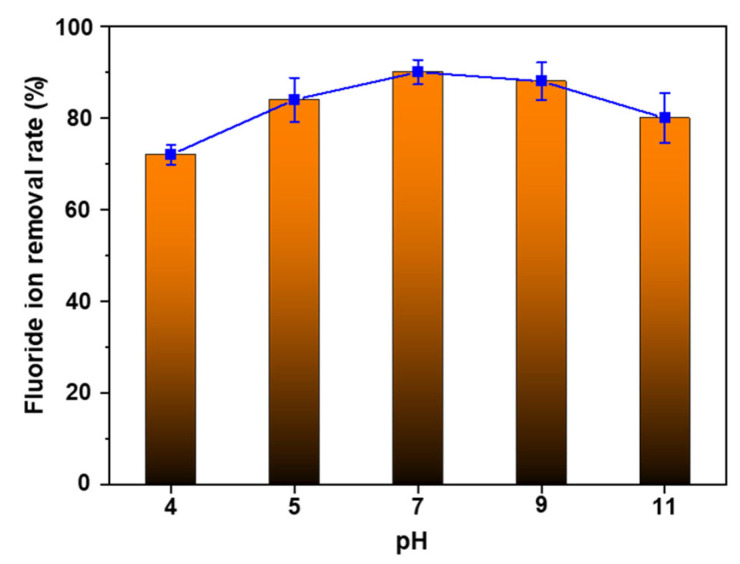
Fluoride ion removal rate of AlOOH-2 under pHs ranging from 4 to 11 (initial fluoride ion concentration: 20 ppm, sample loading: 1.00 g, and adsorption time: 4.0 h).

The pH of the solution is one of the crucial factors in determining the adsorption capacity of the adsorbent. In the semiconductor industry, wastewater can have an acidic pH of about 4 to 5 or an alkaline pH of 11, depending on the specific process involved [47,48]. Therefore, achieving a high fluoride ion removal performance across different pH levels is essential. AlOOH-2’s removal rate of fluoride ions was studied at various pH levels ranging from 4 to 11 (Figure 8). AlOOH-2 exhibited fluoride ion removal rates of 72.6%, 84.1%, 90.0%, 88.4%, and 80.1% at pH 4, pH 5, pH 7, pH 9, and pH 11, respectively. It was observed that the fluoride ion adsorption performance slightly decreased under basic conditions. This behavior aligns with previous studies, which reports that the surface of AlOOH becomes negatively charged at pH levels higher than the point of zero charge (8.5–9.5) of AlOOH determined via zeta potential measurement [3]. This change of charge results in the repulsion of fluoride ions and subsequently reduces adsorption efficiency. On the other hand, the decrease in fluoride ion adsorption performance under acidic conditions appears to be due to the dissolution of the Al-F complex into the solution [49]. Interestingly, AlOOH-2 exhibits relatively consistent performance over a broad pH range, showing a slight decrease of 1.6% from pH 7 to pH 9. This decline is smaller compared to the values (12.0%) reported in a previous study [43]. Also, to evaluate the stability of AlOOH-2 in different pH conditions, dissolved Al concentrations in the simulated wastewater were measured via inductively coupled plasma optical emission spectroscopy (ICP-OES) after the removal of fluoride ions. After this evaluation, it was observed that, in relation to the initial sample dosing amount, only a very small amount of Al was dissolved back into the solution (Table S7).

**Figure 9:**
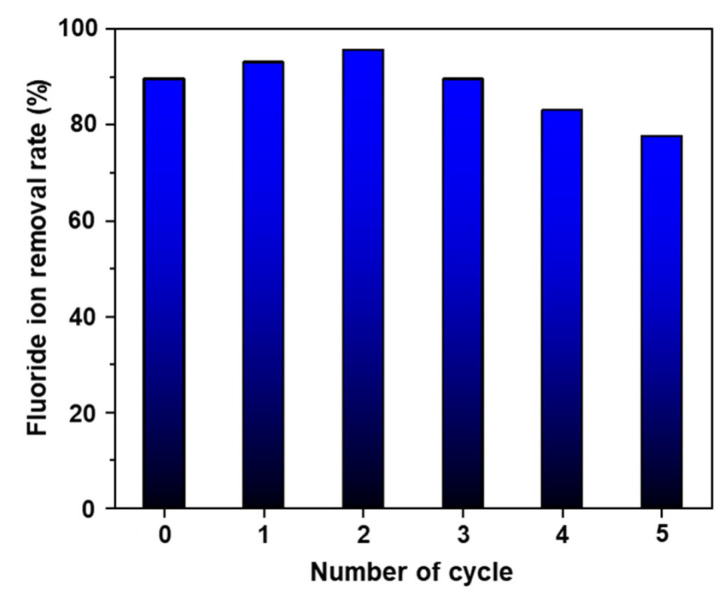
Reusability test for fluoride ion removal of AlOOH-2 through the regeneration process (initial fluoride ion concentration: 20 ppm, sample loading: 1.00 g, and adsorption time: 1.0 h).

Furthermore, the reusability of AlOOH-2 for fluoride ion removal is tested. Initially, AlOOH-2 was tested for its fluoride ion removal rate by soaking it in 25 ml of 20 ppm HF solution for 1.0 h. After fluoride ion removal, the adsorbent was regenerated through immersion of the fluoride ion-adsorbed adsorbent in boiling DI water for 0.5 h. Then, the regenerated AlOOH-2 was tested for its fluoride ion removal rate under the same conditions as the initial test. This regeneration was repeated five times. The results showed that the adsorbent maintained over 80.0% of its removal rate up to the fourth cycle with regeneration, and exhibited around 77.5% of its fluoride ion removal rate in the fifth cycle (Figure 9). It is proposed that the regeneration mechanism involves the exchange of fluoride ions for hydroxyl ions once again during the re-immersion process. This is supported by the decrease in the Al-F peak observed in the XPS analysis following the regeneration process. Furthermore, the concentration ratio of Al-OH/Al-O in the Al 2p spectra exhibited an increase from 1.86 to 2.80. Similarly, in the O 1s spectra, the Al-OH/Al-O-Al ratio showed an increase from 1.70 to 3.59 (refer to Figure S7 and Table S8, in comparison to Figure 6 and Table 1).

**Figure 10:**
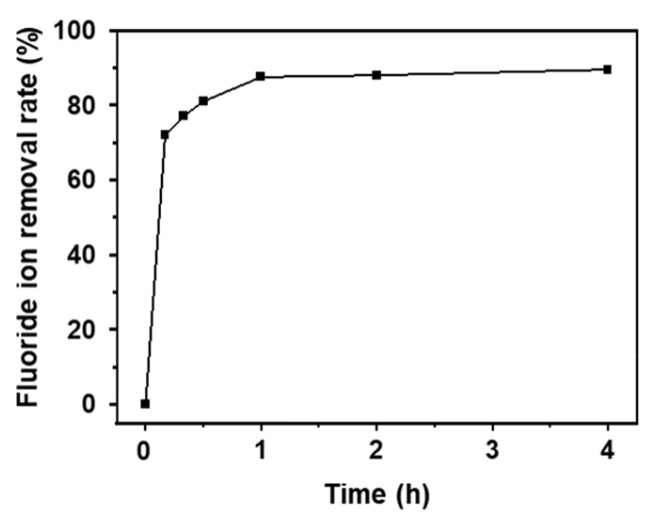
Fluoride ion removal rates of AlOOH-2 in industrial semiconductor wastewater (initial fluoride ion concentration: 20 ppm, sample loading: 1.00 g, and adsorption time: 4.0 h).

The adsorbent developed in this study shows excellent performance in wastewater treatment systems. The fluoride removal rate of AlOOH-2 is specifically evaluated using actual wastewater discharged from the semiconductor manufacturing process (20 ppm). The adsorbent exhibited a rapid removal rate of 72.0% within 10 min, and the rates further increased to 89.5% within 4.0 h (Figure 10), which is a similar performance to that in simulated wastewater (Figure 4a).

**Figure 11:**
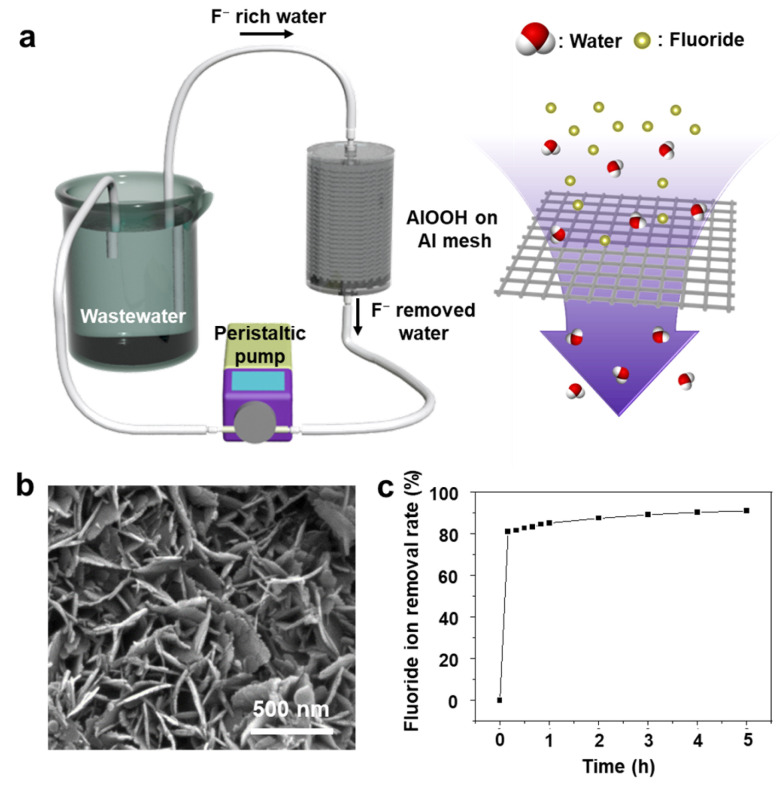
(**a**) Schematic diagram of the flow cell for fluoride ion removal test, (**b**) FE-SEM image of AlOOH on Al mesh, (**c**) fluoride ion removal rates of the AlOOH fabricated Al mesh in HF solution with the flow cell.

Additionally, to examine the possibility of using the AlOOH adsorbent for an actual fluoride ion treatment system, the continuous flow cell operated in a semi-batch mode is adopted, simulating an industrial wastewater treatment module (Figure 11a). To enhance the water flow, Al mesh is utilized instead of Al foil. The AlOOH-fabricated mesh was produced using the same method as for the growth of AlOOH on Al foil (Figure 11b). The fluoride ion removal rate of the AlOOH-fabricated mesh was evaluated in a flow cell using 500 mL of a 20 ppm HF solution at a flow rate of 1 L/min, using 87.56 g of the sample over 5.0 h. Within the initial 10 min, 81.0% of fluoride ions were rapidly removed, and after 5.0 h, a removal rate of 90.9% was achieved (Figure 11c).

## 4. Conclusions

In this work, a cost-effective and eco-friendly synthetic method for producing an AlOOH-based sheet-type fluoride ion adsorbent is successfully demonstrated. The economic advantage is highlighted by the significant cost difference between the utilized aluminum foil and other common Al precursors. The synthesis process is optimized and a high fluoride ion removal rate is achieved by varying the immersion time in boiling water. As a result, AlOOH-2 is obtained, which exhibited an efficient fluoride ion removal rate of 91.0% for fluoride ion concentrations below 20 ppm. Additionally, the cycle performance of the adsorbent is assessed, and its excellent reusability is confirmed through a simple regeneration method involving a re-immersion process in boiling DI water. Importantly, our adsorbent exhibited an efficient fluoride ion removal capability in diverse conditions, including actual semiconductor wastewater and various pH levels. Furthermore, its practical applicability was demonstrated through the use of a continuous flow cell operating in a semi-batch mode. These results highlight the versatility and potential of our adsorbent for various industrial water treatment applications with highly scalable production in the near future.

## Figures and Tables

**Table 1:** Al 2p and O 1s peak parameters for the AlOOH-2 before and after fluoride ion removal.

Electrons	Peak	Binding Energy (eV)	Before Adsorption	After Adsorption
			Concentration (Atomic %)	
Al 2p	Al-O	73.5	31.64	30.53
	Al-OH	74.1	68.36	56.87
	Al-F	75.3	-	12.61
O 1s	Al-O-Al	530.6	31.97	36.96
	Al-OH	532.0	68.03	63.04

## Data Availability

Not applicable.

## Supplementary Materials

The following supporting information can be downloaded at: https://www.mdpi.com/article/10.3390/nano13212838/s1, Figure S1: photographs and cross-sectional view FE-SEM images of AlOOH on Al foil, which were fabricated by immersing Al foil in boiling DI water with different immersing times; Figure S2: FE-SEM images of AlOOH on Al foil, synthesized by immersing in boiling DI water for 10 s, 30 s, 1 min, and 5 min; Figure S3: atomic ratio of Al and O of AlOOH on Al foil with different immersing times in boiling DI water, from EDS analysis; Figure S4: fluoride ion removal rates of AlOOH-2 with different adsorption times and initial fluoride ion concentration; Figure S5: fluoride ions adsorbed by AlOOH-2 with different adsorption time and initial fluoride ion concentrations; Figure S6: FT-IR spectra of AlOOH-2; Figure S7: XPS survey spectra and XPS spectra of F 1s, Al 2p, and O 1s of regenerated AlOOH-2; Table S1: comparison of the costs of common Al salts and Al foil used as precursors for Al-based adsorbent synthesis; Table S2: atomic percent of Al and O of AlOOH on Al foil with different immersing times in boiling DI water via EDS analysis; Table S3: atomic percent of Al and O of AlOOH-0.5, AlOOH-1, AlOOH-2, and AlOOH-4 via spot EDS analysis from a cross-sectional view; Table S4: kinetic model parameters of AlOOH-2 for pseudo-first-order model obtained from sorption experiments with different initial fluoride ion concentrations; Table S5: calculated parameters for Langmuir isotherm models obtained from equilibrium sorption experiments; Table S6: adsorption capacities of various Al-based adsorbents for fluoride ions [3,22,45,46,50,51,52,53,54,55,56,57,58,59]; Table S7: concentrations of dissolved Al in the simulated wastewater with different pH conditions measured using inductively coupled plasma optical emission spectroscopy (ICP-OES) after removal of fluoride ions.; Table S8: Al 2p and O 1s peak parameters for the regenerated AlOOH-2.
