# Supplementary material for: Efficient Fluoride Wastewater Treatment Using Eco-Friendly Synthesized AlOOH
Source: Nanomaterials (Basel). 2023 Oct 26;13(21):2838. doi: 10.3390/nano13212838 (PMC10648790; doi:10.3390/nano13212838)
Supplement: Supplementary file 1 [file nanomaterials-13-02838-s001.zip › nanomaterials-2676121-supplementary FC done.pdf]

Efficient Fluoride Wastewater Treatment Using Eco-Friendly Synthesized AlOOH

Wan-Tae Kim, Joo-Won Lee, Hong-Eun An, So-Hye Cho and Sohee Jeong *

Materials Architecturing Research Center, Korea Institute of Science and Technology, Seoul 02792, Republic of Korea; wantaekim@kist.re.kr (W.-T.K.); lee2080@kist.re.kr (J.-W.L.); ahe9930@kist.re.kr (H.-E.A.); sohyec@kist.re.kr (S.-H.C.)
* Correspondence: soheejeong@kist.re.kr; Tel.: +82-2-958-5369

Supplementary Information

Equation of pseudo first-order model and Langmuir isotherm model.

Equation 1 represents the pseudo-first-order kinetic equation, and Equation 2 represents the Langmuir isotherm equation.

$$q_t = q_e(1 - e^{-k_1 t}) \tag{1}$$

$$\frac{1}{q_e} = \frac{1}{C_e K_L q_m} + \frac{1}{q_m} \tag{2}$$

Where qt is the amount of adsorbed fluoride ion at time t. k1 is the pseudo-first-order adsorption rate constant. Ce and qe are the fluoride ion concentration and the adsorption capacity at equilibrium, respectively. qm is the maximum adsorption capacity. KL is Langmuir constant.

FT-IR analysis of AlOOH-2, before and after fluoride ion removal.

Fluoride ion removal mechanism of AlOOH-2 was analyzed by FT-IR spectroscopy (Fig. S6). With comparison of intensity ratio of Al-O-H band at 1063 cm^{-1} and Al-O band at 594 cm^{-1}, their intensity ratio (Al-O-H/Al-O) were 0.99 and 0.81, before and after fluoride ion removal, respectively. After fluoride adsorption, it is found that the peak at 1063 cm^{-1} compare to the peak at 594 cm^{-1} corresponding to the surface hydroxyl groups decreases after fluoride adsorption, indicating that surface hydroxyl groups are involved in the fluoride adsorption process.

Table S1. Comparison of the costs of common Al salts and Al foil used as precursors for Al-based adsorb ent synthesis.

Precursor	Price (USD/kg)*
Aluminum chloride	116.00
Aluminum isopropoxide	76.50
Aluminum nitrate nonahydrate	194.00
Household Al foil	2.10

* Prices are compared based on the listings provided by Alibaba.com (Alibaba Group Holding Ltd.) for aluminum foil, and Sigma-Aldrich Inc. for the other precursors.

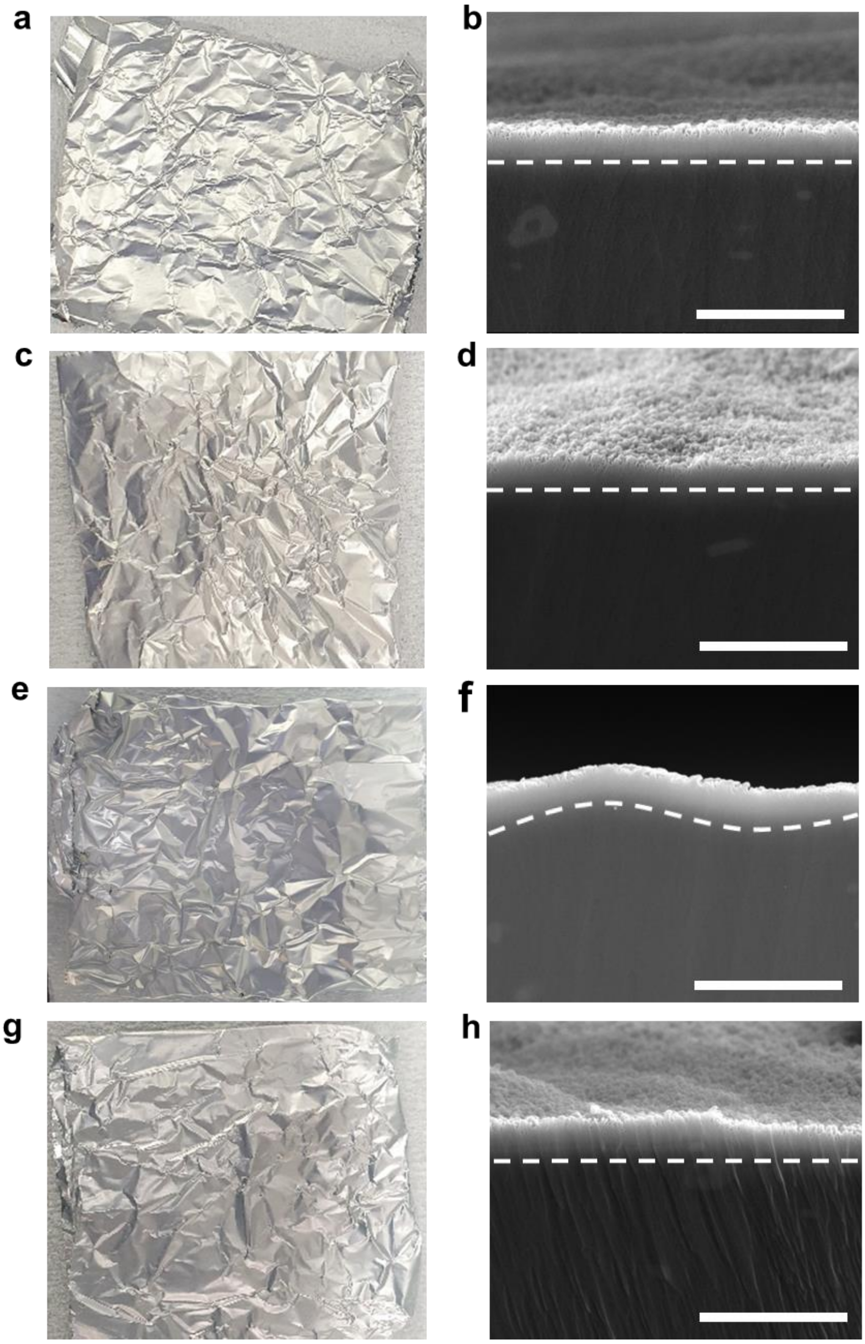

Figure S1. (a, c, e, g) Photographs and (b, d, f, h) cross-sectional view FE-SEM images of AlOOH on Al foil which were fabricated by immersing Al foil in boiling DI water with different immersing time. (a, b : 0.5 h, c, d: 1.0 h, e, f: 2.0h and g, h: 4.0h, white scale bar is 3 μm)).

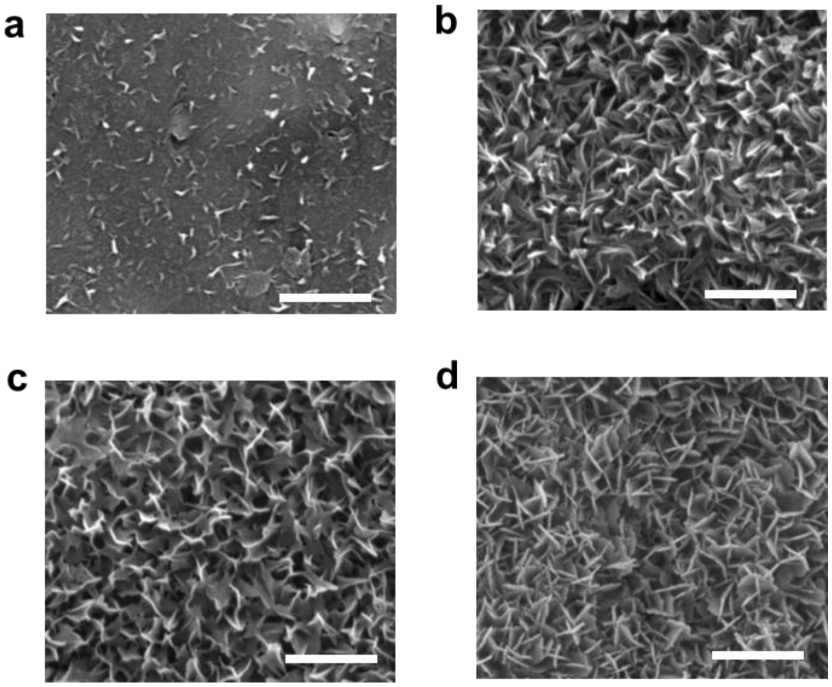

Figure S2. FE-SEM images of AlOOH on Al foil, synthesized by immersing in boiling DI water for (a) 10 sec, (b) 30 sec, (c) 1 min, (d) 5min (refer to AlOOH-10s, AlOOH-30s, AlOOH-1m, AlOOH-5m, white scale bar is 500 nm).

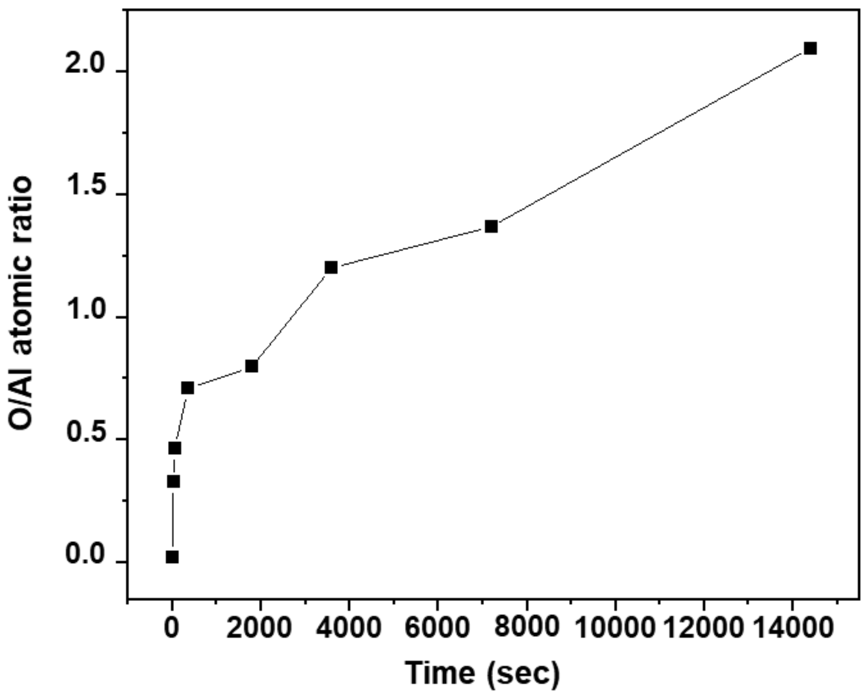

Figure S3. Atomic ratio of Al and O of AlOOH on Al foil with different immersing time in boiling DI water, from EDS analysis.

Table S2. Atomic percent of Al and O of AlOOH on Al foil with different immersing time in boiling DI water by EDS analysis.

Immersing time / Atoms (at %)	10 sec	30 sec	1 min	5 min	0.5 h	1.0 h	2.0 h	4.0 h
O	2.14	24.61	31.63	41.45	44.28	54.53	57.73	67.67
Al	97.86	75.39	68.37	58.55	55.72	45.47	42.27	32.33

Table S3. Atomic percent of Al and O of AlOOH-0.5, AlOOH-1, AlOOH-2, and AlOOH-4 by spot EDS an alysis from cross-sectional view.

Sample / Atoms (at %)	AlOOH-0.5	AlOOH-1	AlOOH-2	AlOOH-4
O	65.55	65.40	66.27	66.39
Al	34.45	34.60	33.73	33.61

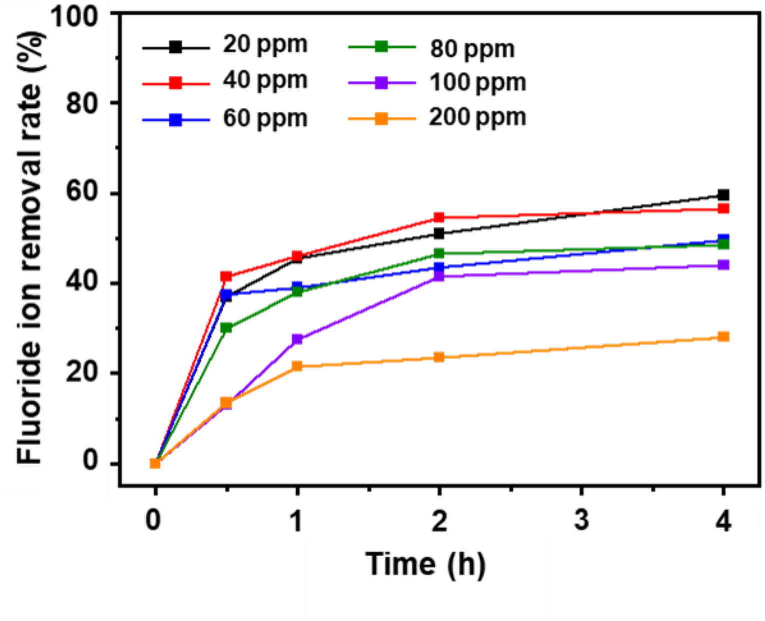

Figure S4. Fluoride ion removal rates of AlOOH-2 with different adsorption time and initial fluoride ion c oncentration (initial fluoride ion concentration: 20 ppm - 200 ppm, sample loading: 0.25 g, and adsorption time: 4.0 h).

Table S4. Kinetic model parameters of AlOOH-2 for pseudo first-order obtained from sorption experiment s with different initial fluoride ion concentration.

Initial concentration (mg/L)	Pseudo first-order model		
	q_e (mg/g)	k_1 (1/h)	R^2
20	0.28	1.04	0.87
40	0.54	1.76	0.95
60	0.68	1.35	0.94
80	0.96	1.49	0.98
100	1.18	0.85	0.98
200	1.35	1.41	0.98

Table S5. Calculated parameters for Langmuir isotherm models obtained from equilibrium sorption experi ments.

Langmuir model		
**qm (mg/g)**	**KL (L/mg)**	**R^2**
1.98	0.02	0.99

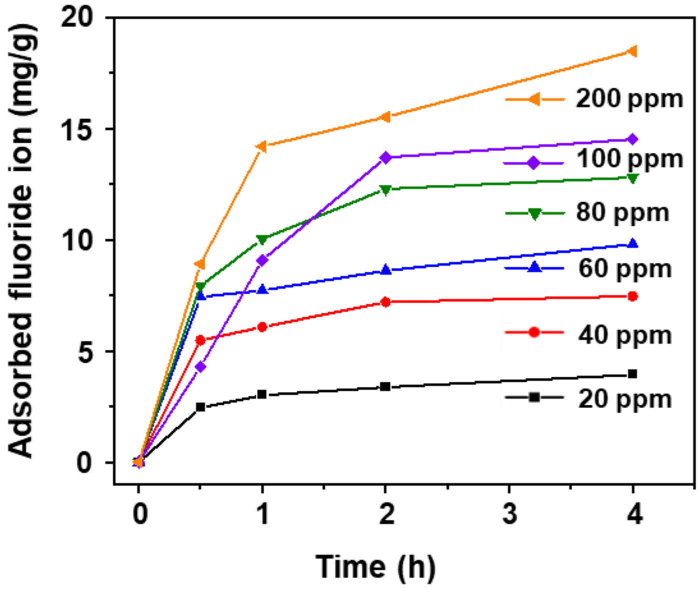

Figure S5. Adsorbed fluoride ion with different adsorption time and initial fluoride ion concentration by A lOOH-2 (This adsorbed fluoride ion value was calculated by the volume ratio calculated from the thicknes s for the AlOOH-only portion of the AlOOH on Al sample, initial fluoride ion concentration: 20 ppm - 20 0 ppm, sample loading: 0.25 g, and adsorption time: 4.0 h).

Table S6. Adsorption capacities of various Al-based adsorbents for fluoride ions.

Adsorbents	Adsorption Capacity (mg/g)	references
Al-modified magnetite ore	1.51	[50]
Al/Fe oxide-loaded tea waste	18.52	[51]
Al_2O_3-modified expanded graphite	5.75	[52]
γ-AlOOH@chitosan shell@Fe_3O_4	67.5	[53]
mesoporous $CoAl_2O_4$	14.8	[54]
Al_2O_3 nanoparticles	9.73	[55]
MgO-loaded Al_2O_3	37.35	[56]
bayerite/boehmite	56.8	[57]
amorphous AlOOH	41.9	[3]
Activated alumina	16.34	[58]
Hydrous-manganese oxide-coated alumina	7.09	[59]
Nano-AlOOH	3.259	[22]
Aluminium hydroxide impregnated macroporous polymeric resin adsorbent	36.61	[45]
Al–Zr loaded tea waste adsorbent	17.54	[46]
AlOOH-2	1.98<br>26.12*	This study

* This value was calculated based on the volumetric ratio, calculated from the height of the AlOOH film in the sample.

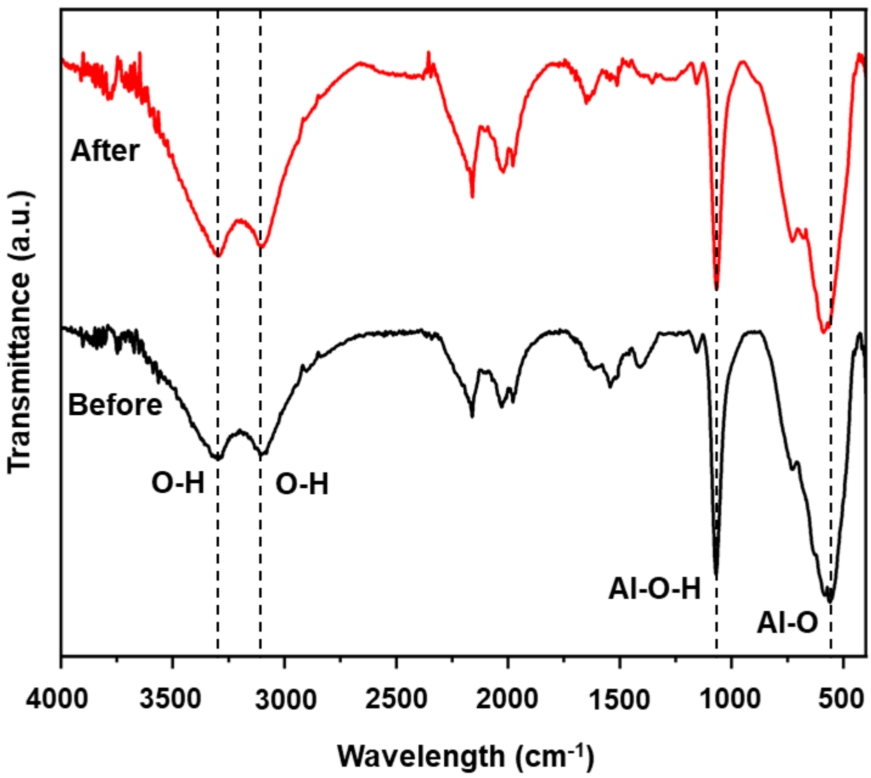

Figure S6. FT-IR spectra of AlOOH-2 (black: before fluoride ion removal, red: after fluoride ion removal).

Table S7. Concentrations of dissolved Al in the simulated wastewater with different pH conditions measured by inductively coupled plasma optical emission spectroscopy (ICP-OES) after removal of fluoride ion. (Relative weight percentage of the initial sample dosing amount)

pH	4	5	7	9	11
Al Conc. (wt%)	0.0191	0.0079	0.0002	0.0035	0.0115

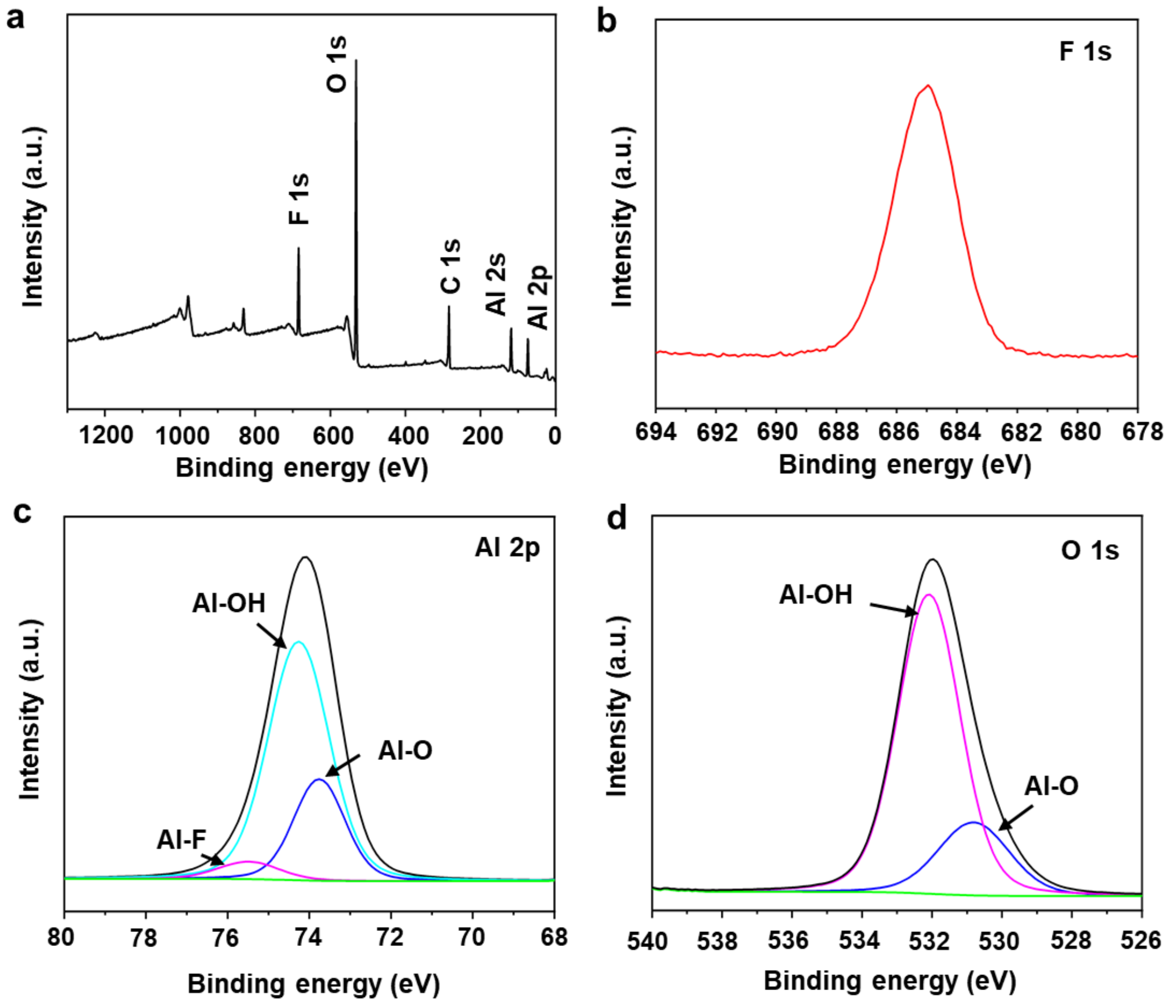

Figure S7. (a) XPS survey spectra and XPS spectra of (b) F 1s, (c) Al 2p and (d) O 1s of regenerated AlOOH-2.

Table S8. Al 2p and O 1s Peak Parameters for the regenerated AlOOH-2.

Electrons	Peak	Binding energy (eV)	Concentration (%)
Al 2p	Al-O	73.5	24.98
	Al-OH	74.1	69.89
	Al-F	75.3	5.13
O 1s	Al-O-Al	530.6	21.78
	Al-OH	532.0	78.22
